# Potential links between the microbiota and T cell immunity determine the tumor cell fate

**DOI:** 10.1038/s41419-023-05560-2

**Published:** 2023-02-24

**Authors:** Amir Hossein Mohseni, Sedigheh Taghinezhad-S, Vincenzo Casolaro, Zhongwei Lv, Dan Li

**Affiliations:** 1 Department of Immunology, University of Pittsburgh School of Medicine, Pittsburgh, PA 15261 USA; 2 Department of Nuclear Medicine, Shanghai Tenth People’s Hospital, Tongji University School of Medicine, Shanghai, China; 3 Department of Medicine, Surgery and Dentistry “Scuola Medica Salernitana”, University of Salerno, Baronissi, Salerno, Italy; 4 Clinical Nuclear Medicine Center, Tongji University School of Medicine, Shanghai, China; 5 Imaging Clinical Medical Center, Tongji University School of Medicine, Shanghai, China; 6 Department of Nuclear Medicine, Sun Yat-sen Memorial Hospital, Sun Yat-sen University, Guangzhou, China

**Keywords:** Immunoediting, Immunological memory

## Abstract

The central role of the microbiota as a pivotal factor regulating anti-tumor immune responses has recently been appreciated. Increasing evidence has put a spotlight on the connection of microbiota to T cells, by showing impaired effector and/or memory responses in germ-free (GF) mice or in the presence of dysbiotic communities, and association with tumor growth and overall survival (OS). These observations also have significant implications for anti-tumor therapy and vaccination, suggesting that the communication between T cells and the microbiota involves soluble mediators (microbiota-derived metabolites) that influence various functions of T cells. In addition, there is growing appreciation of the role of bacterial translocation into the peritumoral milieu from the intestinal tract, as well as of locally developed tumor microbial communities, spatially separated from the gut microbiota, in shaping the tumor microbiome. Collectively, these findings have added new support to the idea that tonic inputs mirroring the existence of tumor microbiome could regulate the function of tumor-infiltrating T cells and tissue-resident memory T (TRM) cells. In this review, we focus on recent advances and aspects of these active areas of investigation and provide a comprehensive overview of the unique mechanisms that play a pivotal role in the regulation of anti-tumor immunity by the microbiota, some of which could be of particular relevance for addressing problems caused by tumor heterogeneity. It is our hope that this review will provide a theoretical foundation for future investigations in this area.

## Facts

- A straightforward link between local/distant immune signatures and the diversity of gut microbiota can be associated with a favorable or unfavorable T cell immune response in a number of gastrointestinal and non-gastrointestinal cancers.
- Microbiota-derived metabolites guide the metabolic rewiring of CD8^+^ T cells toward promoting/inhibiting anti-tumor immunity.
- Gut microbiota can translocate and reside in tumor niches, shape the tumor microbiome landscape, and play a role in the CD8^+^ T cell education within the TME, which together control tumor growth.
- There is a connection between the microbiome at other mucosal sites and the development of local tumors and patient outcomes, independent of the gut microbiota.

## Open question

- How to identify a unique subset of the human microbiota (novel oncomicrobiotics (OMBs)) with great biotherapeutic potential in the induction of CD8^+^ T cell responses among different ethnic groups?
- How do microbiota components instruct T cell responses, contribute to tumor regression and improve patient survival?
- Which mechanisms are involved in the modulation of tumor immunosuppression by local tumor microbial signatures?

## Introduction: leading role of the microbiota in tumor immunity

Cancer immunology research over the past years in mice and human patients, has shown a remarkable progress in identifying the host microbiome as a powerful regulator of host immune responses to multiple solid tumors including colon, lung, pancreas, melanoma, gastric, prostate, and breast [1–5]. The most comprehensive studies have been conducted on the gut microbiome that has a fundamental role in the development of the immune system. In addition, gut microbiota can translocate to and inhabit tumor niches exerting a modulatory role even in tumors at distal sites. Microbes in combination with host factors can therefore define tumor behavior and predict outcome independent of the tumor genomic composition [6].

The advent of omics technologies has allowed discovery of new aspects of the microbiome, and its effects on T cell immunity and cancer, identifying the presence of both negative and positive regulatory bacteria that are capable either of interfering with or enhancing the efficacy of cancer therapy [7]. Landmark studies have highlighted the driving role of the microbiota in regulating several aspects of cytotoxic CD8^+^ T cell functions, and the extensive consequences of these interactions for eliminating tumor cells and promoting cancer control [8–13]. Research shows that in early stages of life intestinal colonization results in microbial antigens trafficking from the intestine to the thymus via the migration of intestinal dendritic cells (DCs) that then stimulate the microbiota-specific T cells expansion [14]. In 2007, for the first time, the beneficial role of the gut microbiota was demonstrated in protocols of adoptive T cell transfer, where commensal strains were shown to stimulate the activation of antigen-presenting cells (APCs) in a lipopolysaccharide (LPS)–Toll-like receptor 4 (TLR4) signaling-dependent manner [15]. Similarly, in immunomodulatory regimens and platinum-based anti-cancer therapies, bacteria-associated TLR4 agonists were shown to modulate tumor necrosis factor (TNF)- and reactive oxygen species (ROS)-mediated anti-tumor effects of tumor-infiltrating myeloid-derived suppressor cells (MDSCs) [16]. The significance of the microbiota on T cell education was seen in germ-free (GF) and antibiotic-treated mice that showed the presence of distinct intestinal T cell receptor (TCR) repertoires in comparison with mice having a normal composition of gut microbiota, indicating that microbial antigens change the development of T cells [17]. Introducing antigen-activated CD8^+^ T cells into GF mice caused weaker overall recall responses and significantly but moderately reduced production of interferon‐gamma (IFN-γ) compared to specific-pathogen-free (SPF) mice. This was suggested to happen mainly due to the absence of microbiota and the subsequent reduction in the pool of memory cells that survive the contraction phase [8]. This observation is in agreement with recent studies supporting the crucial role of tumor-infiltrating tissue-resident memory T cells (TRM) in reaching long-lasting and potent anti-tumor immune responses against solid tumors [18–21] and their association with prolonged survival and a CD8^+^CD103^+^ T cell phenotype [22]. This was especially seen in patients receiving the antibody blocking the inhibitory receptor programmed cell death protein 1 (PD-1) [23]. Using immunogenomic approaches, it was documented that CD8^+^ T cells isolated from cancer patients’ blood could exert their reactivity against predicted neoepitopes almost 3 years after resection of the tumors [24], recommending a straightforward link between memory T cells and the gut microbiota that together participate in achieving a favorable clinical outcome. In other examples, memory responses by IFN-γ–secreting CD8^+^ and CD4^+^ T cells specific for *Akkermansia muciniphila*, *Bacteroides fragilis*, and *Enterococcus hirae* were correlated with the desired clinical outcome in patients with cancer [5, 16, 25, 26]. Collectively, these discoveries point to the remarkable impact of the gut microbiota on tumor growth control by modulating CD8^+^ T cells.

Although CD8^+^ T cells are known for their anti-tumor and cytotoxic activity, some findings in mice proposed that alterations in the composition of the gut microbiota (dysbiosis) were linked to chronic activation of intestinal lamina propria (LP) T cells that could also exert their pathogenic role mostly through stimulating chronic inflammation. This in turn could affect tumor growth and modulate anti-tumor immunity [9]. For instance, the gut microbiota has an impact on tumor susceptibility and inflammatory responses by affecting the frequencies of intestinal LP immune cells, particularly T helper 17 (Th17) and regulatory T cells (Tregs). More importantly, evidence from mouse studies suggests that dysbiosis increases T cell exhaustion, leading to decreased numbers of CD8^+^IFN-γ^+^ T cells inside the tumor microenvironment and intimately diminished anti-tumor immunity. On the contrary, a balanced microbiota is also likely to counteract T cell exhaustion during inflammation and tumor development in the colon [9]. Thus, it might be worthwhile to search for bacterial communities that not only limit T cell exhaustion to maximize anti-tumor immunity but also promote protective CD8^+^IFN-γ^+^ T cell responses in the tumor microenvironment. Therefore, understanding the mechanisms underlying microbiota–immune interactions is a step toward microbiotherapy of cancer.

## Positive and negative impact of the gut microbiota on T cell immunity in the context of cancer

### The gut microbiota remotely controls cancer progression

**Fig. 1:**
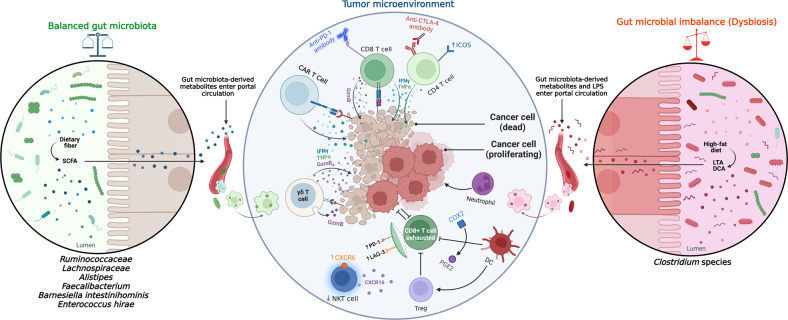
Systemic impact of the gut microbiota on anti-tumor immunity. Interactions between the human microbiome and the immune system can contribute to tumor inhibition and/or tumor progression, via several mechanisms. In a balanced and healthy human gut microbiota and following sufficient dietary fiber intake, bacterial metabolites such as SCFAs derived from gut bacteria can translocate from the gut lumen to blood and eventually regulate T cell immunity. For instance, SCFAs can play an important role in T cell activation by increasing the accumulation and infiltration of CD4^+^, ICOS^+^ and CD8^+^ T cells, enhance the expression of Gzm B, IFN-γ, and TNF-α, improve clinical responses to checkpoint blockade immunotherapies such as anti-PD-1/PD-L1 and anti-CTLA4 antibodies and CAR-T cell therapy, and recruitment of IFN-γ-producing γδ T cells in the TME, all contributing to enhanced anti-tumor immunity. On the opposite side, a gut microbiota imbalance (dysbiosis) induced by high-fat diet, including enrichment of *Clostridium* species, could potentially increase the production of secondary BAs like DCA and microbe-associated molecular patterns, (e.g., LTA and LPS) which can enter the circulation and then up-regulate COX2, induce an increase in PGE_2_, down-regulate CXCL16 production by liver sinusoidal endothelial cells and reduce recruitment of NKT cells, thus further contributing to tumor progression, and inhibit CD103^+^ dendritic cell, IFN-γ, and TNF-α accumulation and enhance tumor Treg accumulation thus contributing to cancer immune evasion. In addition, dysbiotic communities can contribute to hyper-stimulation of CD8^+^ T cells, promoting chronic inflammation and ultimately leading to T cell exhaustion (with expression of PD-1 and LAG-3), and infiltration of neutrophils that can inhibit anti-tumor immunity, which collectively contribute to the development of cancer. SCFA short chain fatty acid, ICOS inducible T cell co-stimulator, Gzm B Granzyme B, IFN-γ interferon gamma, TNF-α tumor necrosis factor α, PD-1 programmed death-1, PD-L1 programmed death-ligand 1, CTLA4 cytotoxic T-lymphocyte associated protein 4, CAR chimeric antigen receptor, γδ T cell gamma delta T cell, secondary BA secondary bile acid, DCA deoxycholic acid, LTA lipoteichoic acid, LPS lipopolysaccharide, COX2 cyclooxygenase-2, PGE2 prostaglandin E2, CXCL16 CXC motif chemokine ligand 16, NKT cell natural killer T cell, LAG-3 lymphocyte activation gene-3, DC dendritic cell.

The connections between the gut microbiota and anti-tumor T cell immunity are in part mediated by gut microbiota-derived metabolites. Short-chain fatty acids (SCFAs), such as acetate, propionate, and butyrate, as products of bacterial fermentation of indigestible polysaccharides, are a prime example of these metabolites that upon transport into the portal circulation via colonocytes, contribute to the regulation of host immunity **(**Fig. 1**)**. Recent studies have demonstrated that specific microbiota-derived SCFAs interact directly with CD8^+^ T cells, causing enhanced IFN-γ-mediated responses in vitro and in vivo [27], and increased CD8^+^ T cell priming in mice [28], and subsequently improve the capacity of stimulated CD8^+^ T cells to differentiate into memory cells with an improved recall capacity in vitro and in vivo [29]. In the light of these findings, in vivo investigations of the effects of fiber-rich diet on immune responses defined by network analysis showed that species of the *Ruminococcaceae* family are fiber-fermenting bacteria producing significant amounts of SCFAs that can contribute to the accumulation of T cells in the tumor, such as CD4^+^ T cells, inducible T cell co-stimulator (ICOS)^+^ T cells, and CD8^+^ T cells, and engage pathways of T cell activation which mediate their anti-tumor effects [30]. In line with these data, another study by Broadfield et al. showed that SCFA-producing *Ruminococcaceae* as well as *Lachnospiraceae* and *Alistipes* are increased following oral administration of metformin (Met) in high-fat diet (HFD)-fed mice, resulting in suppressed growth of MC38 murine colon cancer cells [31]. In addition, these findings were validated by transferring fecal microbiota (FMT) from HFD-Met fed mice to drug naïve, conventional HFD-fed mice, leading to decreased tumor proliferation following expansion in circulating butyrate and propionate. From a mechanistic point of view, metformin administration and the subsequent elevation in the amount of circulating SCFAs could lead to down-regulation of highly activated T cell clusters (CD8^+^, NK1.1^+^, Ki67^+^) in tumors of mice transplanted with fecal microbiota from HFD-Met mice and decreased tumor growth. These data imply that microbiota-derived metabolites guide the metabolic rewiring of stimulated CD8^+^ T cells and mediate metabolic changes in CD8^+^ T cells promoting anti-tumor immunity. Considering the potential effects of SCFAs on tumor invasiveness, further mechanistic studies are awaited to explore the effect of SCFAs on host immune cells and decipher their relative impact on the survival, growth, or activity of tumors.

### Gut microbiota and anti-cancer therapeutics

Growing evidence supports the importance of microbial communities as a therapeutic target in the response to therapy and tumor growth control [32, 33]. However, there are only a handful of known commensal strains isolated from therapy-responsive patients and/or healthy patients that can potentially be used to modulate the functions of host physiological responses and promote anti-tumor phenotypes when transplanted into GF mice [4, 5]. For example, just limited populations of gut bacteria strains, enriched in *Ruminococcaceae*, *Alistipes*, *Parabacteroides*, and *Bacteroides* from healthy human donor feces, are capable of robustly inducing the accumulation of IFN-γ-producing CD8^+^ T cells in the gut, enhancing anti-cytotoxic T-lymphocyte-associated protein 4 (CTLA-4)- and anti-PD-1-mediated tumor growth control [34]. The connection between CD8^+^ T cells and the microbiota also provides a new interpretation to the recent observation that the relative abundance of particular SCFA-producing microbiota constituents is strictly associated with responsiveness to PD-1 blockade in cancer patients [2, 4, 5]. The findings of two newly published clinical trials indicated that SCFA-producing bacteria such as *Lachnospiraceae*-enriched FMT enhanced anti-PD-1 immunotherapy and augmented CD8^+^ T cell infiltration in melanoma patients [35, 36].

Up to now, the U.S. Food and Drug Administration (FDA) has approved four chimeric antigen receptor (CAR) T cell therapies for the treatment of CD19^+^ hematologic malignancies [37–39]. Despite the promising results, disease relapse and CAR-mediated toxicities are still experienced in a wide variety of patients. Although only few studies have looked at the impact of the gut microbiota on CAR-T treatment, very recent elegant studies by Smith and colleagues have shown an association between the therapeutic responses in patients receiving CD19 CAR-T cells and higher abundance of *Faecalibacterium*, *Bacteroides*, and *Ruminococcus* among the gut microbiota. In contrast, increased toxicity and worse survival are associated with exposure to broad-spectrum antibiotics before CAR-T cell infusion in patients with B-cell malignancies. Mechanistically, the nonoxidative branch of the pentose phosphate pathway was enriched in patients with reported toxicities while peptidoglycan biosynthesis was increased in patients who demonstrated a 100-day complete response [40]. Taken together, these results highlight the definite correlation between local immune signatures, such as T cells as potential effectors of anti-tumor immune responses, and the diversity of gut microbiota that in turn can be associated with a favorable response in patients who respond well to treatment.

It has been acknowledged that gut microbiome diversity is correlated with greater response rates in cancer patients treated with radiation therapy. This theory was supported by Sims et al., who demonstrated that distinct gut microbiome compositions, as well as higher diversity, could be seen in patients with prolonged clinical survival [41]. Further investigation on the relative abundance at baseline by using linear discriminant analysis (LDA) showed a significant enrichment of *Enterobacteriales*, *Escherichia Shigella*, and *Enterobacteriaceae* in long-term survivor samples and a significant enrichment of *Dialister*, *Porphyromonas*, and *Porphyromonadaceae* in short-term survivor fecal samples. By phenotyping immune cells isolated from cervical tumor brush samples, the same group also reported an association between expanded CD4^+^ tumor infiltrating lymphocytes (TILs), favorable response upon radiation treatment, and high microbial diversity. These results supported the idea that patients having a more diverse gut microbiota at baseline may benefit from chemoradiation (CRT). However, further investigation of this area of knowledge is needed to understand how alterations in the human gut microbiome following CRT can affect patients’ susceptibility to treatment-associated toxicity. In addition, research imply that the delicate mutualistic symbiosis among the LP-resident immune system and intestinal epithelial cells can be modified by anti-cancer therapeutics, contributing to reprogram anti-cancer immunity. For instance, translocation of some strains of Gram-positive bacteria from the gut lumen to the splenic and mesenteric immune tissues can be induced in vivo by the chemotherapeutic drug cyclophosphamide (CTX) [42]. It was determined that the therapeutic efficacy of CTX would be impaired by extensive antibiotic use unless Gram-positive bacteria are orally gavaged [16]. Building on these results, Daillère et al. by performing ex vivo and in vivo experiments recognized that two intestinal oncomicrobiotics (OMBs), named *Barnesiella intestinihominis* and *Enterococcus hirae*, orchestrate the anti-cancer therapeutic effects of CTX therapy by increasing the recruitment of IFN-γ-producing γδ T cells and CD8^+^ effector TILs, and reducing γδ T17 and Treg cells in cancer lesions [16]. In addition, a recent study by Fluckiger et al. found that in mice bearing *Enterococcus hirae* carrying a specific prophage, which contains a major histocompatibility complex (MHC) class I–binding epitope in the tail length tape measure protein (TMP), a TMP-specific-H-2Kb–restricted CD8^+^ T-lymphocyte response would be mounted upon treatment with CTX or immunotherapy with anti-PD-1 antibodies. More interestingly, they linked the long-lasting benefit of immunotherapy with anti-PD-1 antibodies in patients having renal and lung cancer with the tumor expression of a TMP–cross-reactive antigen and existence of the enterococcal prophage in feces, indicating that delivery of engineered bacterial strains with the ability to express a TMP epitope may result in more effective immunotherapy in mice [43]. Consequently, finding a unique microbial signature in patients having cancer and its alterations during immunotherapy, radiotherapy, and chemotherapy can offer further insight into the mechanisms involved in the modulation of immune responses and contribute to improving the outcomes of treatment in these patients.

### Connection between gut microbiota and tumor progression

Beyond the impact of the gut microbiota on control of cancer progression, a definitive role of gut bacteria in tumorigenesis has been proposed. The era of microbiotherapy has come of age with the emergence of inflammatory disorders initiated with an overt deviation of the gut microbiota [16]. Dysbiotic communities able to disrupt epithelial barrier function are thought to be provoking factors in neoplastic transformation [44]. During tumor progression, the entrance in circulation of bacteria may be permitted by leaky vasculature in disorganized tumors, and an immunosuppressed environment may offer a refuge for them [45, 46]. Notably, specific metabolites from dysbiotic species actively modulate innate immunity as well as adaptive immunity signaling [47]. It is accepted that microbial dysbiosis in mouse models of various cancers can change the tumor microenvironment (TME) through enhancing immune surveillance, raising sensitivity to immunotherapy, and provoking the over activation of T cell (Fig. 1). As a result, imbalanced interactions between a dysbiotic gut microbiota and the host immune system have been connected to a number of gastrointestinal and non-gastrointestinal cancers, including cancers of the colon, liver, and pancreas [9, 48, 49].

Two major risk factors for some cancers, including colorectal cancer (CRC), are type 2 diabetes and obesity [50]. In response to HFD, metabolic endotoxemia due to increased levels of circulating gut microbiota-derived LPS in obese individuals plays an important role in related metabolic diseases and progression of inflammation, suggesting a link between nonalcoholic fatty liver disease (NAFLD) pathogenesis and the gut microbiota-derived endotoxin. A vast body of evidence supports the actual role of NAFLD, along with the associated microbiome signature, in the development of hepatocellular carcinoma (HCC). Due to the global pandemic of type II diabetes mellitus (T2DM) and obesity, liver cirrhosis as a consequence of NAFLD (NAFLD-cirrhosis) ranks as the most common liver disease around the world, and NAFLD-associated HCC (NAFLD-HCC) is projected to be the leading cause of HCC. Animal and human research are now endeavoring to uncover the role of metabolite as well as microbiome signatures in shaping and regulating immune responses and HCC pathogenesis [48, 51, 52]. For instance, Behary et al. demonstrated that dysbiosis of the gut microbiota, including enrichment in *Ruminococcaceae* and *Bacteroides* members, in patients with NAFLD-HCC is correlated with SCFAs production (particularly butyrate), leading to reduced CD8^+^ T cells and increased Treg cell frequencies [53]. Overproduction of deoxycholic acid (DCA) and lipoteichoic acid (LTA), as secondary bile acids (BAs) following HFD, due to certain species of *Clostridium*, led to acceleration of carcinogen-induced liver cancer progression in mice through overexpression of cyclooxygenase-2 (COX-2) (rate-limiting enzyme in the prostaglandin cascade) and stimulation of senescence in hepatic stellate cells. It has been shown that expression of TNF-α and IFN-γ by LTA-stimulated liver immune cells can be suppressed by prostaglandin E_2_ (PGE_2_), indicating that in obesity-related HCC PGE_2_ through enhancing the prevalence of Treg cells and inhibiting the accumulation of CD103^+^ DCs may impair the anti-tumor functions of CD8^+^ T cells [51]. In another study, *Clostridium* species following BAs production, including murine-specific muricholic acid or lithocholic acid (LCA), caused down-regulation of chemokine (C-X-C motif) ligand 16 (CXCL16) in liver sinusoidal endothelial cells (LSECs), which contributed to diminished recruitment of natural killer T (NKT) cells to the liver, resulting in the development of tumor in diverse mouse models of primary or metastatic liver cancer [48].

Since the dysbiotic microbial communities may act as an important contributor to oncogenesis in different malignancies and perturbation of the normal immune system in cancer patients is linked to the intestinal tract, it can be postulated that removing specific metabolites derived from dysbiotic communities could be sufficient to down-regulate the growth of tumors and promote the accumulation of T cells. Along this line, Ma et al. reported that dietary administration of primary BAs or depletion of *Clostridium* by oral vancomycin augmented the expression of CXCL16 in LSECs and led to higher accumulation of IFN-γ-producing NKT cells, enrichment of CXCR6^+^CD62L^low^CD44^hi^ effector memory CD4^+^ and CD8^+^ T cells, and enhancement of tumor control in mice [48]. This possibility was also raised in studies of pancreatic ductal adenocarcinoma (PDAC), in which the activation of CD8^+^ T cells along with down-regulation of MDSCs and up-regulation of M1 macrophage differentiation and T helper 1 (Th1) differentiation of CD4^+^ T cells, as documented by elevated expression of T-box transcription factor 21 (TBX21, also known as T-Bet), TNF-α, and IFN-γ, was favored by bacterial ablation, resulting in alleviation of PDAC tumor burden [54]. Furthermore, the outcomes of studies in patients having PDAC indicate that targeting the microbiome by administering oral antibiotics, and the subsequent gut bacterial ablation, in combination with checkpoint-directed immunotherapy might be a promising approach for experimental therapeutics [54]. Collectively, these results re-emphasized a potential role for microbiome reversal as a therapeutic target in the regulation of tumor-directed immune responses.

## Positive and negative impacts of the tumor microbiota on T cell immunity in the context of cancer

### The tumor microbiome

It has been determined that microorganisms also reside in sites outside of the gut and other portals of entry [52, 55, 56], particularly in tumors, where they represent underappreciated environmental agents and can exert regulatory functions on the TME immune and inflammatory program [44]. Up to now, a tumor microbiome has been characterized in many tumor types, such as pancreas cancer [49, 57], prostate cancer [58], breast cancer [59], melanoma [60], gastric cancer [61], thyroid cancer [62, 63], renal cell carcinoma (RCC) [64], nasopharyngeal carcinoma (NPC) [65], lung cancer [66, 67], and cutaneous T cell lymphoma (CTCL) [68, 69] (Table 1); Nevertheless, because of its low biomass, the characterization of the intratumor bacterial colonies has remained challenging. More recently, comprehensive analysis of more than 1500 tumor microbiomes across seven cancer types, using a mixture of assays like next-generation sequencing coupled with immunohistochemistry (IHC) and RNA fluorescence in situ hybridization (FISH), showed that (i) in many human solid tumors, LPS and bacterial RNA and DNA can be detected; (ii) intratumor bacteria are mostly intracellular and are present in both cancer and immune cells; (iii) a more diverse and richer microbiome than seen in other tumors is found in breast tumors, including live bacteria from three main phyla *Firmicutes*, *Actinobacteria*, and *Proteobacteria*; (iv) distinct microbial compositions are present in different tumor types, whereby the microbiome of pancreatic cancer is dominated by *Proteobacteria*; *Fusobacterium nucleatum* was also reported as a dominant bacterial species in pancreatic and breast tumors; the most represented taxa in non-gastrointestinal tumors were species from the *Actinobacteria* phylum such as *Micrococcaceae* and *Corynebacteriaceae* families; and the most abundant bacteria in colorectal tumors belong to the *Bacteroidetes* and *Firmicutes* phyla [70].

**Table 1:** Association of intratumoral bacteria with human tumor.

Tumor type	Number of cancer patients	Intratumor bacteria	Outcome/conclusion	Ref.
Breast tumor	221	*Streptococcus* *Propionibacterium*	(i) Positive correlation between tumor bacteria and T cell activation-related genes. (ii) Provide evidence supporting a role for local microbiome–immune crosstalk in breast cancer. (iii) Delineate breast microbial profiles associated with multiple prognostic clinical variables.	[59]
	33	*Fusobacterium* *Atopobium* *Gluconacetobacter* *Hydrogenophaga* *Lactobacillus*	(i) Breast tissue microbiome is distinct from the overlying breast skin tissue, as well as from skin and buccal swab samples. (ii) Enrichment in the tumor taxa correlated with malignancy. (iii) Significant differences is in the microbial composition of the breast tissue microenvironment in patients with benign versus malignant disease.	[82]
Lung tumor	89	*Acinetobacter* *Halomonas* *Chryseobacterium*	(i) Statistically significant decrease of the percentage of tumor bacteria in the TME compared with adjacent normal tissue samples. (ii) High bacterial load in the tumor combined with increased iNOS expression is a favorable prognostic factor. (iii) High bacterial load combined with the increased number of FOXP3^+^ cells as a marker of poor prognosis.	[67]
	38	*Gammaproteobacteria*	(i) Tumor bacteria correlate with low PD-L1 expression and poor response to checkpoint-based immunotherapy, translating into poor survival.	[66]
Pancreatic tumor	113		(i) Tumor bacteria mediate the expression of a long isoform of the bacterial enzyme CDDL that can metabolize the chemotherapeutic drug gemcitabine into its inactive form and play a critical role in mediating resistance to chemotherapy.	[83]
	27	*Enterobacteriaceae*	(i) Biliary stent placement and neoadjuvant chemotherapy can promote the infiltration and growth of intra-tumor bacteria in the setting of PDAC and lead to to increased chemoresistance.	[84]
	10	*Enterococcus*	(i) Markedly higher abundance of tumor bacteria in patients undergoing preoperative stent placement in the common bile duct can be associated with the development of a postoperative pancreatic fistula.	[85]
	15	*Tepidimonas* *Leuconostoc* *Sutterella* *Comamonas* *Turicibacter* *Streptococcus* *Akkermansia*	(i) More abundant *Tepidimonas* in tumors. (ii) Larger tumors resulted in lower levels of *Leuconostoc* and *Sutterella*. (iii) Increased lymph node metastasis associated with higher levels of *Comamonas* and *Turicibacter* in tumor tissues. (iv) The levels of *Streptococcus* and *Akkermansia* were decreased in tumor tissues in the case of tumor recurrence.	[86]
	187	*Acinetobacter baumannii* *Mycoplasma hyopneumoniae* *Acidovorax ebreus* *Geobacillus kaustophilus* *Escherichia coli*	(i) Presence of several bacteria species within PAAD tumors is linked to metastasis and immune suppression. (ii) Increased prevalence and poorer prognosis of PAAD in males and smokers are linked to the presence of potentially cancer-promoting or immune-inhibiting microbes. (iii) *A. baumannii* and *M. hyopneumoniae* were found to be correlated to smoking-mediated changes in the genome that cause PAAD. (iv) *A. ebreus*, *A. baumannii*, *G. kaustophilus*, and *E. coli* demonstrated differential abundance and activation of cancer and immune-associated pathways in male versus female PAAD patients.	[87]
	62	*Acinetobacter* *Pseudomonas* *Sphingopyxis*	(i) Increasing abundance of tumor microbiome is highly associated with PDAC oncogenesis and the induction of inflammation.	[49]
	68	*Pseudoxanthomonas* *Streptomyces* *Saccharopolyspora* *Bacillus clausii*	(i) The tumor microbiome unique to patients with long-term survival (LTS) is characterized by the recruitment and activation of CD8^+^ T cells to the tumor milieu and might also be a useful predictor of patients’ outcomes.	[57]
Gastric tumor	53	*Methylobacterium*	(i) Intratumoral *Methylobacterium* is inversely correlated with the frequency of CD8^+^ TRM T cells and TGF-β in the TME and linked to poor prognoses in patients with gastric cancer.	[61]
RCC tumor	66	*Actinobacteria* *Proteobacteria* *Firmicutes* *Cyanobacteria* *Bacteroidetes*	(i) Tumor tissue microbiome has lower alpha diversity, compared to the adjacent conventional normal kidney tissue. (ii) Kidney tumors with high content of PU.1^+^ macrophages and CD66b^+^ neutrophils in the stroma were characterized by a lower bacterial burden. (iii) In kidney tumors with high bacterial burden, the number of PU.1^+^ cells and CD66b^+^ neutrophils were associated with a poor prognosis.	[64]
Prostate tumor	16	*Propionibacterium* spp. *Staphylococcus* spp.	(i) *Propionibacterium* spp. were the most abundant and *Staphylococcus* spp. were more represented in the tumor/peri-tumor tissues. (ii) Possible pathophysiological correlation between the composition of the local microbial niche and the presence of the tumor itself.	[58]
Melanoma tumor	447	*Lachnoclostridium* *Gelidibacter* *Flammeovirga* *Acinetobacter*	(i) Intra-tumor-residing gut microbiota could modulate chemokine levels and affect CD8^+^ T cell infiltration, thereby improving patient survival in cutaneous melanoma.	[60]
Thyroid tumor	30	*Sphingomonas* *Aeromonas* *Comamonas* *Acinetobacter* *Peptostreptococcus*	(i) The abundance of *Sphingomonas* and *Aeromonas* was significantly increased in tumor tissues and abundance of *Comamonas*, *Acinetobacter*, and *Peptostreptococcus* was significantly increased in peritumor tissues. (ii) Abundance of *Sphingomonas* was significantly higher in N1 stage than in N0 stage. (iii) Richness and diversity of thyroid microbiota were significantly lower in thyroid tumor samples in comparison with matched peritumor tissues.	[62]
	80	*Pseudomonas* *Rhodococcus* *Ralstonia* *Acinetobacter* *Sphingomonas* *Brevundimonas*	(i) The tumor bacterial diversity in patients with advanced lesions (T3/T4) was significantly higher than that in patients with relatively mild lesions (T1/T2). (ii) Tumor-resident microbiota, albeit at a low biomass, play a key role in promoting PTC progression.	[63]
NPC	802	*Corynebacterium* *Staphylococcus*	(i) Intratumoral bacteria within NPC tissues mainly originated from the nasopharynx. (ii) Intratumoral bacterial load was negatively associated with T-lymphocyte infiltration and led to poor survival in patients with NPC.	[65]
CTCL	6	*Corynebacterium* spp. *Cutibacterium* spp. *Staphylococcus* spp.	(i) Patients with MF and/or SS showed no marked differences in skin viral or fungal communities. (ii) Two *Corynebacterium* species (*C. tuberculostearicum* and *C. simulans*) were increased on MF and SS skin. (iii) MF and SS skin displayed lower relative abundances of *Cutibacterium acnes* and *Cutibacterium namnetense*. (iv) Commensal *Staphylococci* (*S. capitis*, *S. epidermidis*, and *S. hominis*) trended higher in MF. (v) Relative abundances of *C. tuberculostearicum* were high in patients with stage IVA1.	[69]
	7	*Bacillus safensis*	(i) Induce malignant T cell activation and cytokine secretion. (ii) Skin microbiota of patients with CTCL have a potential role as instigators of tumorigenesis.	[68]

*iNOS* inducible nitric oxide synthase, *FOXP3*^*+*^ Forkhead box P3, *PD-L1* programmed cell death ligand 1, *CDDL* cytidine deaminase, *PDAC* pancreatic adenocarcinoma, *PAAD* pancreatic adenocarcinoma, *A. baumannii* *Acinetobacter baumannii*, *M. hyopneumoniae* *Mycoplasma hyopneumoniae*, *A. ebreus* *Acidovorax ebreus*, *G. kaustophilus* *Geobacillus kaustophilus*, *E. coli* *Escherichia coli*, *CD8*^*+*^ *TRM T cell* CD8^+^ tissue-resident memory T cell, *TGF-β* transforming growth factor-beta, *TME* tumor microenvironment, *RCC* renal cell carcinoma, *PTC* papillary thyroid cancer, *NPC* nasopharyngeal carcinoma, *CTCL* cutaneous T cell lymphoma, *MF* mycosis fungoides, *SS* Sezary syndrome.

**Fig. 2:**
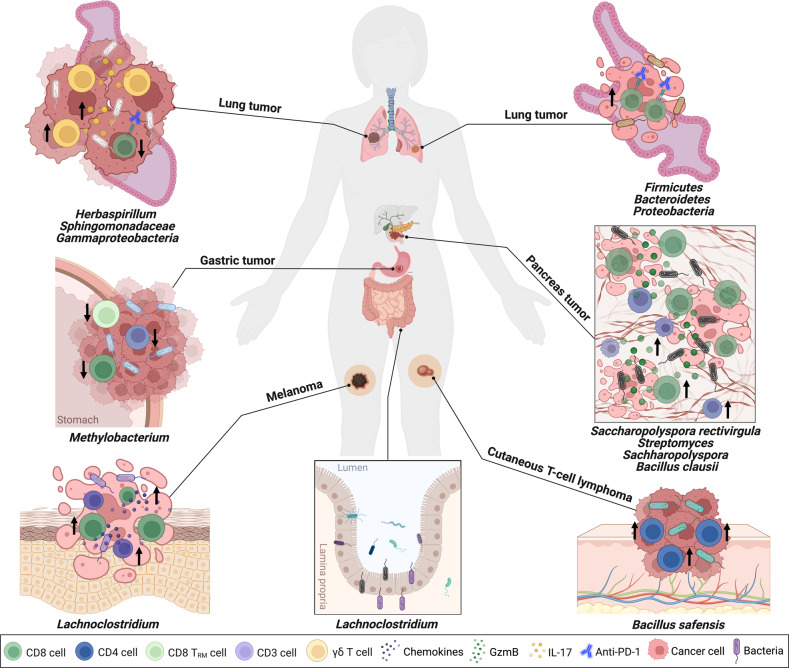
The tumor microbiota may dynamically contribute to the microenvironment reconstruction and lead to cancer pathogenesis or tumor regression and eventually patient survival. Gut: Gut microbiota can exert an effect on tumor immunity both systemically and locally. Bacterial translocation from the gut lumen to the lymph nodes and blood can further shape the local tumor microbiome. Pancreatic cancer: Enrichment of *Saccharopolyspora rectivirgula*, *Streptomyces*, *Sachharopolyspora*, and *Bacillus clausii* can result in tumor infiltration and activation of CD8^+^ T cells and are associated with long-term survival and improved anti-tumor immunity in pancreatic cancer. Melanoma: *Lachnoclostridium* is correlated with infiltration of cytotoxic CD8^+^ T cells and high expression of chemokines CCL5, CXCL10, CXCL9 that collectively contribute to significant increase in patient survival in cutaneous melanoma. Gastric cancer: High abundance of *Methylobacterium* leads to low levels of CD3^+^ and CD8^+^ TILs, an exhausted CD8^+^ TRM phenotype and infiltration as well as decrease in TGF-β expression in the TME of patients suffering from gastric cancer, which collectively contribute to gastric cancer development. Lung cancer: High abundance of *Gammaproteobacteria* in the cancerous lung and their abundance in the tumor surroundings is significantly correlated with low PD-L1 expression that is also partly reflected in poor PFS and a trend toward worse overall survival under ICI therapy. While enrichment of *Firmicutes*, *Bacteroidetes*, and *Proteobacteria* in local NSCLC are associated with improved OS of ICI-treated patients. CTCL: *Bacillus safensis*, by inducing clonal proliferation of skin-homing T cells (such as CLA^+^, CCR4^+^ CD4^+^ T cells) leads to malignant T cell activation and increased STAT3 phosphorylation, which altogether result in tumorigenesis in CTCL patients. CCL5 C-C motif chemokine ligand 5, CXCL10 C-X-C motif chemokine ligand 10, CXCL9 CXC motif chemokine ligand 9, TIL tumor-infiltrating lymphocyte, CD8 TRM tissue-resident memory CD8^+^ T cell, TGF-β transforming growth factor-beta, TME tumor microenvironment, PDL-1 programmed death-ligand 1, PFS progression-free survival, ICI immune checkpoint inhibitor, NSCLC non-small cell lung cancer, OS overall survival, CTCL cutaneous T cell lymphoma, CLA cutaneous lymphocyte‒associated antigen, CCR4 chemokine (C-C motif) receptor 4, STAT3 signal transducer and activator of transcription 3.

Antigen-specific recognition of microbiota by T cells has been explained earlier [71, 72]. These insights will add new dimensions to the notion that tonic inputs mirroring the existence of tumor microbiome can regulate CD8^+^ T cell immune responses within the TME. In addition, the gut microbiota can translocate and reside in tumor niches, where they may play a beneficial role against tumors at distal sites [6], thanks to their ability to activate the immune system, promote systemic anti-tumor immunity and ultimately affect tumor responses to therapies [57]. However, several studies have shown contrasting effects of the tumor microbiome on T cells and inherently the biology of tumors (Fig. 2). Therefore, further investigations and better understanding of the effects of intratumor bacteria on T cell immunity may shed further light on the clinical impact of the tumor microbiome and help design novel therapeutic strategies against tumors.

### The connection of gut microbiota, tumor microbiome, and T cell immunity with overall survival (OS)

It has been reported that most bacterial communities located in the tumoral milieu commonly exist in the gut microbiome, implying that bacteria from the gut can translocate to other tissues and tumors and regulate T cell immune responses against tumors [73]. This hypothesis was empowered in studies associating the exceptional outcome of long-term survivors to T cell immune responses [57, 74, 75], whereby it was proposed that, independent of the genomic composition of the tumor, microbial host factors may regulate the behavior of the tumor and the clinical outcomes. Consistently, tumors with higher densities of cells carrying a Th1 gene signature and CD8^+^ T effector cells are linked to more favorable prognosis [9]. Data showed that PDAC tumors from long-term survivors are enriched in MUC16 neoantigen and elicit more robust activation and infiltration of CD8^+^ T cells. Intriguingly, the neoantigens displayed homology to peptides derived from infectious pathogens, which in turn are identified by the human TCR repertoire, suggesting a molecular mimicry with microbial epitopes [76]. Building on these data, an actual link between gut microbiota, tumor microbiome, T cell immunity, and long-term survival has been discovered recently in a nice study by Riquelme et al., where just a limited number of gut bacteria able to translocate to the tumor and contribute to the tumor microbiome are sufficient to result in prolonged survival in patients with PDAC [57]. In that study long-term survivors exhibited unique diversity and composition of the tumor microbiome that was distinct from that of adjacent healthy pancreatic tissue and associated with more sustained CD8^+^ T cell response along with expression of a high number of Gzm B^+^ cells in the TME. Importantly, enrichment of some species like *Saccharopolyspora rectivirgula*, *Streptomyces*, *Sachharopolyspora*, and *Bacillus clausii* can influence immune infiltration in the TME and is highly predictive of long-term survival in PDAC patients. FMT from healthy controls, short-term PDAC survivors, and long-term PDAC survivors into mice orthotopically implanted with KPC pancreatic cancer cell lines, could modulate the intratumoral microbiome and, eventually, anti-tumor immunity in the recipient mice [57]. Specifically, FMT from long-term survivors into KPC-implanted mice led to activation of CD8^+^ T cells and increased tumor infiltration, greater serum levels of interleukin-2 (IL-2) and IFN-γ, and improved tumor control. Conversely, FMT from short-term survivors into recipient mice resulted in faster tumor growth because of augmented intra-tumoral infiltration of Treg cells and MDSCs. These in vivo data as well as related clinical evidence clearly showed that the gut microbiome could shape the tumor microbiome landscape, in that a strong significant association was found between the tissue densities of CD8^+^ T cells, the control of tumor growth, and the diversity of the tumor microbiome [57].

Confirming these results, characterization of the microbiome of the local non-small cell lung cancer (NSCLC) by employing 16S rRNA gene amplicon sequencing of bronchoscopic tumor biopsies isolated from patients receiving PD-1/PD-L1-targeted immunotherapy showed high proportions of *Bacteroidetes*, *Proteobacteria*, and *Firmicutes* associated with prolonged OS of irrespective of the clinical response to progression-free survival (PFS) or ICI treatment [66]. In addition, Zhu et al., by deep mining RNA-seq data from a large cohort from the Cancer Genome Alta (TCGA), demonstrated that intratumor bacteria are linked to increased cytotoxic CD8^+^ T cell infiltration which associated with prolonged OS in patients suffering from advanced cutaneous melanoma, and significantly lower mortality risk. Their results revealed that gut microbes inhabiting in tumor tissues, such as *Lachnoclostridium* genus, a SCFAs producer, ranked high for positive connection with CD8^+^ T cell infiltration. In fact, a high abundance of intratumor *Lachnoclostridium* was positively linked to the expression of chemokines C-C motif chemokine ligand 5 (CCL5), C-X-C motif chemokine ligand 10 (CXCL10), CXC motif chemokine ligand 9 (CXCL9), which, together, were associated with significantly increased patient survival and reduced mortality in cutaneous melanoma. Consequently, manipulating the gut microbiome (particularly *Lachnoclostridium*) may enrich tumor infiltrating CD8^+^ T cell frequencies and improve patient outcome following immunotherapy [60]. In line with these interesting findings, investigation of the intracellular bacteria-derived peptide repertoire in melanoma lesions using human leukocyte antigen (HLA) peptidomics and 16S rRNA gene sequencing showed that colonizing bacteria can migrate into melanoma cells, after which their non-self HLA-I and HLA-II-restricted peptides can be presented by both APCs and tumor cells. All these processes subsequently provoke robust CD4^+^ and CD8^+^ T cell immune responses, demonstrating that the identical peptide can be a target on tumor cells for immune attack and upon presentation on APCs can start an immune response as well [77]. Further investigation in this area, aimed at precisely discovering the mechanisms of how microbe-related immune modification induce tumor regression and OS, is likely to lead to identification of novel OMBs which are appropriate for use in specific therapeutic combinations.

### Connection between the local microbiota and tumor progression

More recent evidence supports a link between the microbiome at other mucosal sites in the development of local tumors independently of the gut microbiota. For instance, Peng and colleagues, by assessing the tumor microbiota of patients having gastric cancer, found that the relative abundance of *Methylobacterium* was negatively linked to CD3^+^ and CD8^+^ TILs, and in turn associated with poor prognoses. Subsequent experiments in in vivo models and human samples confirmed that higher abundance of *Methylobacterium* associated with an exhausted phenotype in CD8^+^ TRM and lower numbers of infiltrating CD8^+^ TRM cells in the TME of gastric cancer that led to significantly reduced in expression of transforming growth factor-β (TGF-β) in the TME, implying that *Methylobacterium* may have a key role in gastric carcinogenesis [61]. Interestingly, this study did not uncover a link between the genera enriched in the tumor tissues and in the feces of patients with gastric cancer. Therefore, it was proposed that local tumor microbial signatures, independent of the gut microbiota, may specify tumor behavior and patient outcomes. A similar study found that *Rhodococcus*, *Acinetobacter*, *Sphingomonas*, *Ralstonia*, *Brevundimonas*, and *Pseudomonas* were only residing in papillary thyroid carcinoma (PTC) [63] but not in the gut [78].

The possibility of a connection with other mucosal sites was raised when it was found that the lung tumor burden was significantly related to the lung local bacterial burden, but not with the fecal bacterial content. In this study intratracheal administration of a bacterial consortium isolated from late-stage lung tumors into KP mice shortly after tumor initiation led to accelerated tumor progression. Mechanistically, the lung microbiota dysbiosis in this model, characterized by over-representation of various bacterial taxa like *Herbaspirillum* and *Sphingomonadaceae*, induced the production of interleukin-23 (IL-23) and interleukin-1β (IL-1β) from myeloid cells in a myeloid differentiation primary response 88 (MYD88)-dependent manner, leading in turn to lung-resident γδ T cell proliferation and expression of IL-17 and other pro-inflammatory factors to up-regulate local inflammation and eventually tumor cell proliferation. Consistent with in vivo data, PathSeq analysis uncovered a relation between human NSCLC and local dysbiosis, and IHC analysis of patient samples showed enrichment of γδ T cell infiltration in lung adenocarcinoma (LUAD) relative to normal lung tissues. These results offer clear and compelling experimental evidence to support a connection between the local airway microbiota and developing lung tumor growth, mediated by local γδ T cells [79]. Previous studies have supported a link between CTCL, T cell activation and microbiome [68, 80, 81]. For example, Lindahl and co-workers showed a potential link between intravenous (IV) administration of broad-spectrum antibiotics (carbapenem) and a significant decrease in the fraction of malignant T cells in lesional skin colonized by *Staphylococcus aureus* (SA) in patients with advanced-stage CTCL. This phenomenon itself correlated with decreased signal transducer and activator of transcription 3 (STAT3) signaling, expression of interleukin-2 high-affinity receptors (CD25), and cell proliferation in lesional skin, suggesting a critical role of local microbial signature in skin tumorigenesis [80]. In a study investigating the role of the skin microbiome in CTCL patients, Dehner et al. identified a connection between induction of malignant T cell activation and *Bacillus safensis* within CTCL lesions [68]. These data postulated the hypothesis that cutaneous bacteria, such as *B. safensis*, which drive the clonal proliferation of skin-homing, cutaneous lymphocyte-associated antigen (CLA)^+^, chemokine (C-C Motif) receptor 4 (CCR4^+^) CD4^+^ T cells, may also induce the activation of malignant T cells and tumorigenesis in patients with CTCL [68]. Collectively, these data demonstrate that tumor-associated microbiota in cancers proximal to gut like colon and pancreas cancer are possibly derived from the gut microbiota, while the local tumor bacterial composition of more distant tumors like gastric, thyroid, and lung cancer would likely depend on bacterial translocation from oral or nasal sources. However, further investigation is required to confirm the routes of bacterial translocation to many types of tumors.

## Conclusions and future perspectives

Strong and encouraging evidence indicates that the microbiome is a powerful modulator of host immune responses either directly or through metabolites and can exert both anti-tumorigenic and/or pro-tumorigenic functions, thus impacting clinical outcomes and OS in a number of malignancies. Along this line, emerging literature supports the concept that the microbiome can modulate T cell immunity by stimulating T cells and promoting recall responses by memory CD8^+^ T cells, emphasizing its fundamental role in determination of the repertoire of T cells in the TME and tumor cell fate. Here, we highlighted a link between host microbiome signatures and T cell responses that influence tumor immunity and propose some important questions to be tackled in future studies in the field. Nevertheless, only sparse studies implicate this link and studies to date are not sufficient to uncover the entire picture of the mechanisms mediating the interactions of tumor-infiltrating immune cells with gut and more so tumor microbiome. In addition, the significance of the microbiota in cancer metastasis and recurrence has yet to be experimentally validated. The discovery of particular taxa within the gut microbiota and/or the local bacterial burden that substantially alter host responses via immunomodulation and associate with prolonged OS may have important implications in the clinical management of these cancers. Additional studies along this line, including well-powered clinical studies in large-scale cohorts, are warranted to better define the role of microbiota-induced T cells in the treatment of different types of cancer and help design novel therapeutic strategies.

## Data Availability

Data sharing is not applicable to this article as no new data were created or analyzed in this study.
